# Social ecological resources for youths living with HIV in western Uganda

**DOI:** 10.3389/fpsyg.2023.1176754

**Published:** 2023-08-15

**Authors:** Sofie Vindevogel, Emmanuel Kimera

**Affiliations:** ^1^Department of EQUALITY//Research Collective, Hogeschool Gent, Ghent, Belgium; ^2^Department of Public Health, Mountains of the Moon University, Fort Portal, Uganda

**Keywords:** HIV, youth, resilience, photovoice, empowerment, resources

## Abstract

**Introduction:**

The adversities faced by youths living with HIV (YLWH) are manifold, resulting not only from the health impact but also from society’s response to HIV and the people living with it. This study sought to explore these youths’ perceptions and representations of what promotes resilience.

**Methods:**

Photovoice methodology was chosen to elicit first-person accounts that are grounded in lived experience and experiential knowledge. Eleven young people, boys and girls aged 14–21 living in western Uganda, participated in seven group sessions aimed at imagining, producing and discussing visual stories about what fosters resilience in the face of HIV-related adversity. The visual stories were subjected to inductive content analysis by the participants, and then thematically analyzed and interpreted by the researchers using the theoretical framework of social-ecological resilience.

**Results:**

We found that participants experience well-being amidst HIV-related adversity through managing tensions in material resources, sense of identity, power and control in their lives, cultural adherence, relationships, sense of cohesion and social justice.

**Discussion:**

The findings add to the body of knowledge on youth resilience in Sub-Saharan Africa by documenting multisystemic resources for YLWH in Ugandan communities. The findings further show that resources are highly incidental and situational, neither widely available nor structurally embedded in society. The study therefore informs the global HIV/AIDS agenda to spur ecologies of resilience around YLWH.

## 1. Introduction

The Human Immunodeficiency Virus (HIV) and its associated disease, the Acquired Immunodeficiency Syndrome (AIDS), are profoundly impacting the lives of many youths globally, who tend to be predominantly situated on the African continent. Research has started to document the manifold challenges of living with HIV for youths in Sub-Saharan Africa (Lowenthal et al., 2014; Vreeman et al., 2017; Woollett et al., 2017; Kimera et al., 2020a). This has contributed to an enhanced understanding of HIV not only as a health issue, but also as a condition that affects the interrelated life domains of those afflicted with it as well as the families, communities and other social-ecological systems that surround them.

Our previous research in Uganda documented that adversities and challenges reported by youths living with HIV (YLWH) are not only due to the impact of HIV on their health, but are typically compounded through interactions with these surrounding systems (Kimera et al., 2020a,b). Although social attitudes and public discourses have evolved considerably since the emergence of the pandemic in the 1980s, they are shown to be still predominantly negative, stigmatizing and disempowering in Uganda (Obare et al., 2011; Nabunya et al., 2020; Kimera et al., 2020b; Kimera et al., 2021) and elsewhere (Sangowawa and Owoaje, 2012; Mburu et al., 2014; Dahlui et al., 2015; Asamoah et al., 2017; Crowley et al., 2021; Adams et al., 2022). These social conditions create additional, everyday adversities for these youths, leading to self-devaluation (Filiatreau et al., 2021), occupational deprivation (Kimera et al., 2020a) and discrimination (Sangowawa and Owoaje, 2012), among other consequences that jeopardize their well-being.

Other dimensions of the lives and social ecologies of YLWH can protect against this impact and facilitate well-being. Such dimensions have generally received limited scientific attention (Theron and Van Breda, 2021a,b), creating a dearth of knowledge on what enables resilience to HIV-related adversity in Sub-Saharan Africa. The few studies available have mainly focused on individual resources, noting the importance of young people’s psychological attributes and coping mechanisms, such as psychological adjustment (Perez et al., 2021), health promoting behaviors (Harper et al., 2019) or prosocial behaviors (Bhana et al., 2016; Woollett et al., 2017).

Attention to the multisystemic drivers of resilience is more recently on the rise (Dulin et al., 2018; Crowley et al., 2021; Shevell and Denov, 2021; Theron and Van Breda, 2021a). This emerging research points to the importance of resources such as peer support (Mark et al., 2019), neighborhood social capital (Dulin et al., 2018), alongside specialized care and support (Fournier et al., 2014). However, there is still limited understanding of the resources that matter to YLWH and how these foster resilience in the face of HIV-related adversity. Especially resources within the social environments, which can offer the first level, sustainable, and immediate redress for YLWH’s well being, have received less scholarly attention. This study adds to this body of research by exploring the social ecological resources that strengthen young people to deal with HIV-related adversity and challenging living conditions.

Drawing on the theoretical perspective of social ecological resilience, as developed by Ungar, this study focusses on “both the capacity of individuals to navigate their way to health-sustaining resources, including opportunities to experience feelings of well-being, and a condition of the individual’s family, community and culture to provide these health resources and experiences in culturally meaningful ways” (Ungar, 2008, p. 225). Resilience processes are thus conceived of as the interaction between the person’s actions in seeking resources that enable experiences of wellbeing in the face of adversity and the contextual responsiveness to provide or negotiate access to such resources.

Based on cross-cultural research, Ungar and Liebenberg (2011) locate resources for young people in three main areas: the individual, relationships with primary caregivers, and the broader spiritual, educational and cultural context. Ungar (2008) also identified seven tensions youths typically have to navigate in experiencing resilience (i.e., access to material resources, relationships, identity, cohesion, power and control, cultural adherence, and social justice).

Resonant with this framework, we sought to centralize YLWH’s analyses and perspectives on social ecological resources that promote resilience in the face of HIV. This aimed at understanding and documenting the youth’s unique ways of dealing with HIV related challenges in their daily life, and the social ecological responses that allow them to experience feelings of wellbeing. Previous research, using photovoice methodology with YLWH in western Uganda, has demonstrated how the centralization of lived experiences generates localized understandings, expanding dominant ideas about YLWH and what helps them best (Fournier et al., 2014).

In addition, we aimed to capture and disseminate empowering self-representations of YLWH as an antidote to the detrimental effects of stigma and social disqualification of people with HIV. When their unique, idiosyncratic viewpoints are given the necessary platform, they can prove to be shared and socially constructed, broadening the scope from individual experiences to social ecologies (Liebenberg, 2018). Participatory methods that aim at engaging marginalized or underrepresented perspectives in research, such as photovoice, can provide such a platform for young people to express and discuss their views with stakeholders, and ultimately stimulate social change and resilient responses at different socio-ecological levels (Suffla et al., 2012; Lofton et al., 2020). The research itself can then become an empowering practice that stimulates critical consciousness, affirms the voice and agency of those involved, challenges harmful hegemonic discourses, and calls for social justice (Seedat et al., 2015).

## 2. Methods

### 2.1. Participants and setting

The target group was young people, defined by the WHO as 10–24 years old, both male and female, who had been identified as HIV positive and also were aware of their serostatus. The study was conducted at a regional referral hospital in Kabarole district, western Uganda. This hospital is one of the major HIV treatment centers in the district and runs a weekly youth Anti-Retroviral Therapy clinic. The clinic also organizes a peer support group. Eleven youths aged 14–21 years were recruited from this peer support group. Selection was purposive based on the inclusion criteria of: being aged 10–24 years, being aware of the HIV status, having been on therapy for not less than 6 months, willingness to participate in multiple sessions of group and individual discussions, and being able to speak either English or the local languages Lutooro or Luganda. Exclusion was applied to potential participants who were physically and mentally unwell basing on the health workers’ assessment. Priority was given to participants who were previously involved in a similar study with the same research team, documenting HIV-related adversities and challenges (reference published article). Those unavailable or unwilling to participate were replaced by other members of the peer support group. During the selection, efforts were made to include both male and female participants with varied ages.

### 2.2. Photovoice

This study was part of a larger participatory action research project. For this study, the photovoice method was chosen to provide youths with an opportunity to represent themselves and their lives. Participants were invited to analyze and represent in their photos and accompanying narratives the social ecological resources that to them speak of resilience and allow them to experience wellbeing in the face of HIV. Giving them a camera centralized their perspectives, moving them from the margins to the center of knowledge construction (Van Wolvelaer et al., 2021). This not only gave the participants control over how they were represented but also potentially opened up new ways of seeing YLWH who are typically underrepresented or misrepresented by the outsiders’ perspective (Jarldorn, 2019). In addition, we pursued meaningful engagement throughout the research and sought to engender a sense of ownership in participants, which is imperative to photovoice methodology (Wang, 2006). Photovoice had been used successfully with young people in Uganda and these previous studies (Fournier et al., 2014; Seedat et al., 2015; Kimera et al., 2020b) provided important insights and reflections that helped us to refine the research protocol for this study.

### 2.3. Data collection

The study protocol was reviewed and approved by the Research Ethics Committee of the AIDS Support Organization (TASO) in Uganda (reference number, TASOREC/009/18-UG-REC-009) and the Uganda National Council of Science and Technology (reference number SS 4587). We also sought permission to conduct this study from the hospital management and the youth antiretroviral therapy clinic. We contacted potential participants by telephone. In a private quiet room at the clinic, each potential participant was given information about the research project and then asked for their assent or consent to participate. In the case of minors (aged 14–17 in this project), informed consent was also obtained from their legal representatives. We also obtained consent to audio-record and photo release so that the dialogues could be quoted and the photos could be published or exhibited. We ensured that the identities of the participants were not revealed in the photos, transcripts and reports. Self-chosen pseudonyms are used throughout this article.

Seven consecutive sessions were organized. The first session aimed to establish rapport and introduce participants to the concept and procedure of photovoice. During this session, participants were also acquainted with representing themselves through photographs. The second session focused on resilience and guided participants to adopt a resilience lens to identify and appreciate the resources that strengthen them. As there is no vernacular idiom for resilience in the context of western Uganda, the working definition of resilience was “being able to cope with or overcome challenges related to living with HIV, because of individual and community strengths that allow you to experience feelings of wellbeing”.

In the third session, the participants conceived and visualized images representing the resilience-enabling resources in the form of hand-drawn pictures as precursors for the photographs. At the end of this session, each participant was given a digital camera to take photographs documenting resilience in their everyday lives. They carried the camera with them and had the opportunity to take photographs until the next session. Accompanying narratives were either written down in a notebook or recorded audially by the participants.

In the fourth and fifth sessions, batches of photographs were received from participants. The photos were extracted from the cameras into a computer by the researchers and stored in different folders clearly marked with each participant’s pseudonym. Participants were then asked privately to select photos they wanted to present in a group discussion and those they wanted to present to researchers only. All participants choose to present their selected photos in a group discussion. These two sessions were thus designed to create a safe space for participants to share their photos and narratives. Participants took turns presenting their photos using a laptop and projector provided by the researchers. The non-presenting participants were always invited to share their reflections on this presentation, and to guide the presenting participants in selecting the photos that the group most related to and found most empowering.

In the sixth and seventh sessions, participants discussed their selected photos around the questions “What does it reveal about what strengthens me in dealing with challenges in my life because of HIV? What does it say about how resources (people, places, things, …) can support me and can promote my wellbeing? Why is this an empowering image and how can this lead to more empowering representations of YLWH?” To stimulate group reflection, we used a variation of the “SHOWeD” technique, a well-established technique for facilitation photovoice discussions (Wang and Redwood-Jones, 2001): What do you See here? What is Happening? How does this relate to our lives? Why does this strength exist? What can we do to harness it? We aimed to create optimal conditions for dialogue and to implement the practice of speaking with, rather than for, others. The sharing of photos in this group allowed participants to move from critical self-reflection of lived experiences to an understanding of how resilience is fostered.

### 2.4. Data analysis

At various instances throughout the photovoice process, participants were stimulated to make meaning and co-construct interpretation of the photos and accompanying narratives in light of the central research question. They did this first at the level of their personal data, and later in group at the level of the data generated by all participants. As such, they carried out preliminary data analysis during the discussion of photos.

It involved the inductive identification and formulation of themes represented in participants’ photos and accompanying narratives. The researchers facilitated a discussion about what the data meant to the youths, creating space for them to express their thoughts in terms that were familiar and meaningful in that context. Once they had clustered photos that spoke to similar and shared understandings of resources, they were invited to choose a name or description for each cluster, which then became an initial theme. In the results section, these themes are indicated by single quotation marks. Based on this preliminary inductive content analysis by participants, subsequent analysis was undertaken by the researchers based on their transcripts of group discussions, the photos and their captions, and the researchers’ field notes. Thematic analysis was conducted based on the themes identified by the participants in relation to the resilience literature, which resulted in latent themes clustering the participants’ initial themes.

We drew on the resilience literature developed by Ungar et al. (2007) and Ungar and Liebenberg (2011). This is further elaborated in the results section, where the study’s findings are situated in relation to tensions in resilience identified and validated on the basis of cross-cultural research (Ungar, 2008). Nevertheless, we have taken into account the contextually specific ways in which resources become meaningful and resilience is understood and observed (Ungar and Liebenberg, 2011; Vindevogel et al., 2015) as well as the notion of resilience as “a dynamic state of tension between and among individuals, families, communities and their culture” (Ungar et al., 2007, p. 301).

As these thematic areas and tensions should be understood as interlocking and intersecting, they are not discussed separately but are highlighted from the data in this study. By letting the data speak, a more integrative and contextually relevant analysis can ensue. In our experience, taking the time to work closely with these young people created a rich discursive space for deconstructing and understanding resilience in the face of living with HIV in Uganda.

Following these sessions, the participants were actively involved in determining strategies for research valorization, whereby the generated insights are translated into actions that benefit society. The sharing of photos and accompanying narratives beyond the safe space of the research group has the potential to elicit critical consciousness, critical dialogue and knowledge production, and ultimately social change (Wang, 2006; Jarldorn, 2019). The youths proposed various action plans to reach various audiences and influence different socio-ecological levels, including taking the visual narratives to schools and organizing debates with students to raise their awareness of the (social and institutional) challenges and resources for YLWH. Another action plan involved the publication of a photo book, with participants deliberately selecting the photos and accompanying stories they felt were important to share with a wider audience. Engaging them in devising action plans for their visual stories and included messages to influence their society for the better gave them a sense of agency for social change and unleashed their potential as change agents (Suffla et al., 2012), as illustrated in the findings section.

## 3. Findings

### 3.1. Material resources

Some of the themes generated by participants relate to the availability of material resources that can facilitate feelings of wellbeing: “treatment to improve health”, “farming for food and income”, “means of transport”, “knowledge about health”, and “engage in income generation”. Ungar et al. (2007, p.296) also refer to “the availability of structural provisions, including financial assistance and education, as well as basic instrumental needs, such as food, shelter and clothing, access to medical care and employment”.

The prominence of such – seemingly basic, everyday – material resources in the participants’ photos and narratives suggests that such resources are not self-evident to them. In addition to living in a resource-limited country, the youths’ narratives make clear that it is often even more difficult for YLWH to make ends meet and fulfil instrumental needs. Limitations in material resources lead to restrictions in other areas of life. As a result, documenting the importance of material resources from the lived experience acquires significance for understanding resilience in this context.

For Unique Unice, for instance, being given a bicycle and choosing to ride one in defiance of prevailing gender norms helped her access health care and experience feelings of wellbeing:

“This small bike (on the photo) is like a good friend to me. It used to be very hard for me to walk all the way from home to come here to the clinic for my appointments. But since my uncle bought me this bike, I ride fast and get here in a short time. In my area it is not common for girls to ride bikes, but for me, I do not mind because those who say that cannot give me money for the *boda boda* (passenger motorbike).” – Unique Unice, 18-year-old girl

**Figure 1:**
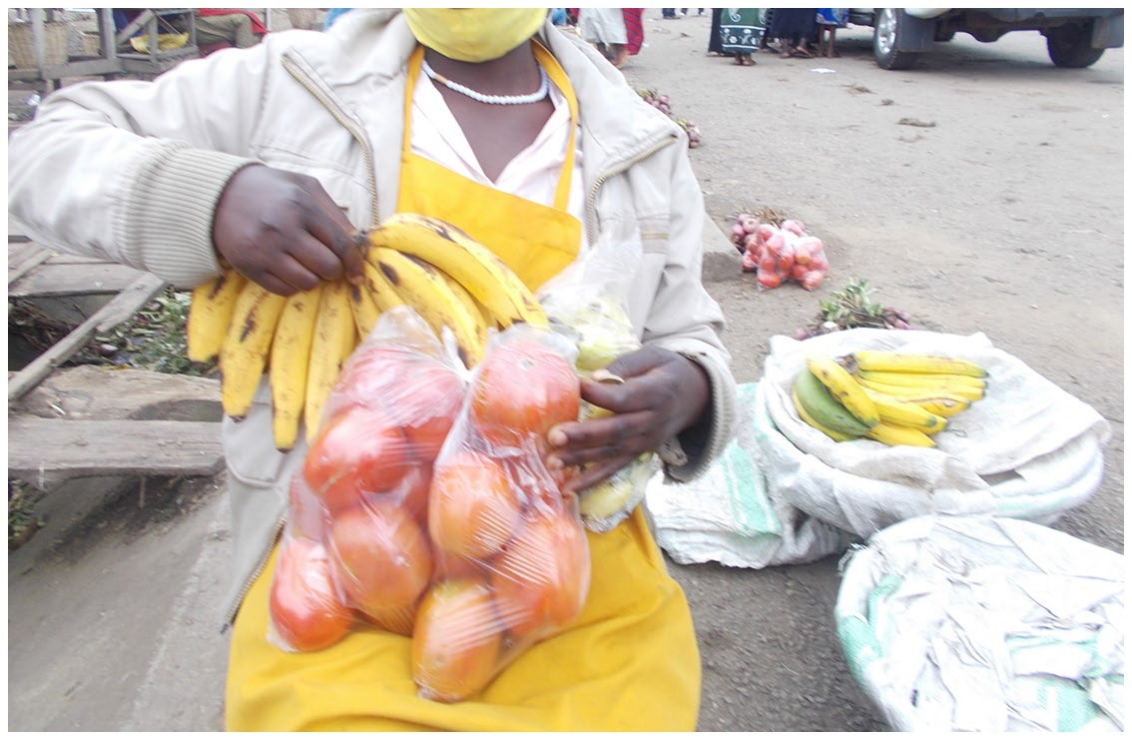
“Instead of coming to the streets to beg, I come to make money” Maggie, 19-year-old girl.

Maggie was left to look after herself, and showed in her photographs (e.g., Figure 1) how she had become self-reliant rather than dependent on others for material resources:

“I started working in the market when my mother died and I could no longer go to school. My business gives me money for transport to the clinic and to buy other things I need as a girl because there is no one else to buy for me. In the evenings I come to the streets to sell fruits and vegetables to motorists. I do not care what others say about me because I know no one can look after me. Instead of coming to the streets to beg, I come to make money.” – Maggie, 19-year-old girl

The mere fact that such material resources were available – either self-generated or provided by others – was seen as valuable, but also the experience that these resources in turn facilitated access to other resources and helped to navigate tensions in other areas was repeatedly emphasized. In particular, when material resources supported “being self-reliant”, participants found them helpful in resolving tensions around identity as well as power and control. These are discussed in the next sections.

### 3.2. Identity

HIV has a profound effect on the youths’ sense of identity and self-image. Ungar and Liebenberg (2011, p.136) conceptualize identity as “personal and collective senses of purpose, self-appraisal of strengths and weaknesses, aspirations, beliefs and values, including spiritual and religious identification.” In interactions with their social environment, they often see their identity confined to being a seropositive person. HIV then becomes a ground for dehumanization, disqualification, and rejection. The participants’ sense of identity was strongly influenced by the dominant trope of YLWH as weak, suffering and vulnerable people.

**Figure 2:**
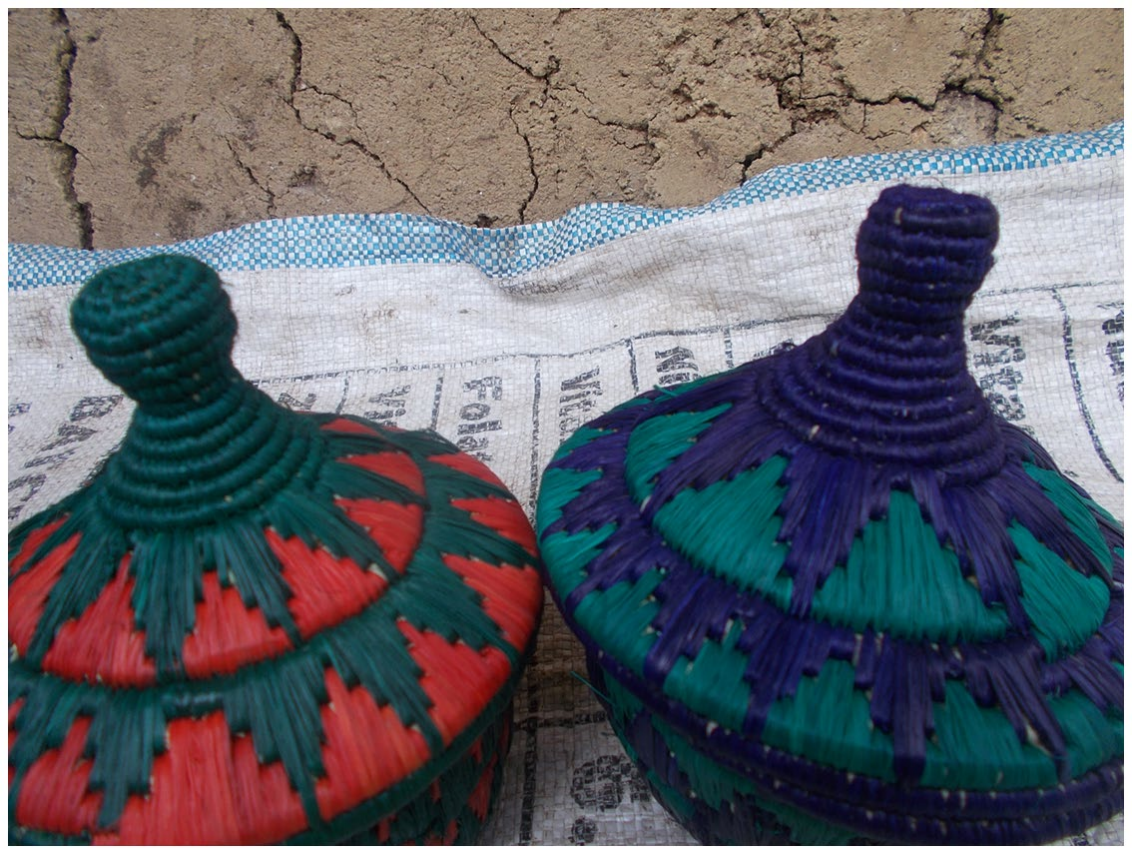
“I make these baskets in my free time to keep myself busy and not to think about the HIV in my life.” Passion, 17-year-old girl.

“Doing something you like” was collectively identified as an important theme, expressing how resources helped YLWH to cultivate a sense of self and purpose. As echoed in the following quotes, resources empowered participants to experience and embody a sense of themselves as competent, capable, talented and strong people (See Figure 2).

“When my father died, he left this land for me and the neighbours thought I was going to sell it because they know I am sick (HIV positive) and they thought I was also going to die. They were also saying that I am weak and I cannot do any farming. I have shown them that they were wrong because I did not die and I did not sell the land. I am now growing maize and yams on this land, which I sell in town to get money to take care of all my needs. With my own little money I am building this small house for my family. I am very happy because many people who are older than me and do not have HIV cannot build their own houses.” – King, 17-year-old boy

“I make these baskets in my free time to keep myself busy and not to think about the HIV in my life. I sell the baskets at 30,000 Shillings each to people who use them to serve *kaalo* (millet meal) or for decoration. It is good for young people with HIV to learn to work with their hands and not to see themselves as needy people or weak people who cannot work.” – Passion, 17-year-old girl

The search for a positive sense of self and the practice of “loving yourself” were also seen as facilitating relationships with others and gaining relational support, as discussed further. “In life we choose what we want” says Unique Unice. “If you have HIV and you do not love yourself then who will love you? But when you love yourself, you take good care of your life and you look good, then others will also love you.” – Unique Unice, 18-year-old girl.

### 3.3. Power and control

Participants also struggled with tensions in experiencing power and control in their lives, after a period of profound loss following the HIV diagnosis. Ungar and colleagues define “power and control” as “the capabilities within, and the resources surrounding, the participants to experience material and/or discursive power in terms meaningful to their context” (Ungar et al., 2007, p. 298).

The youths repeatedly emphasized how they sought to disprove disempowering discourses and representations of YLWH and to deal with disenfranchisement. Junior, a 14-year-old male participant, also framed one of his photos in relation to disempowering representations and approaches to YLWH in society by stating: “I want to show that even though people call us ‘sick children’, we can live life like other children. We can take care of ourselves and do chores like other children at home.”

**Figure 3:**
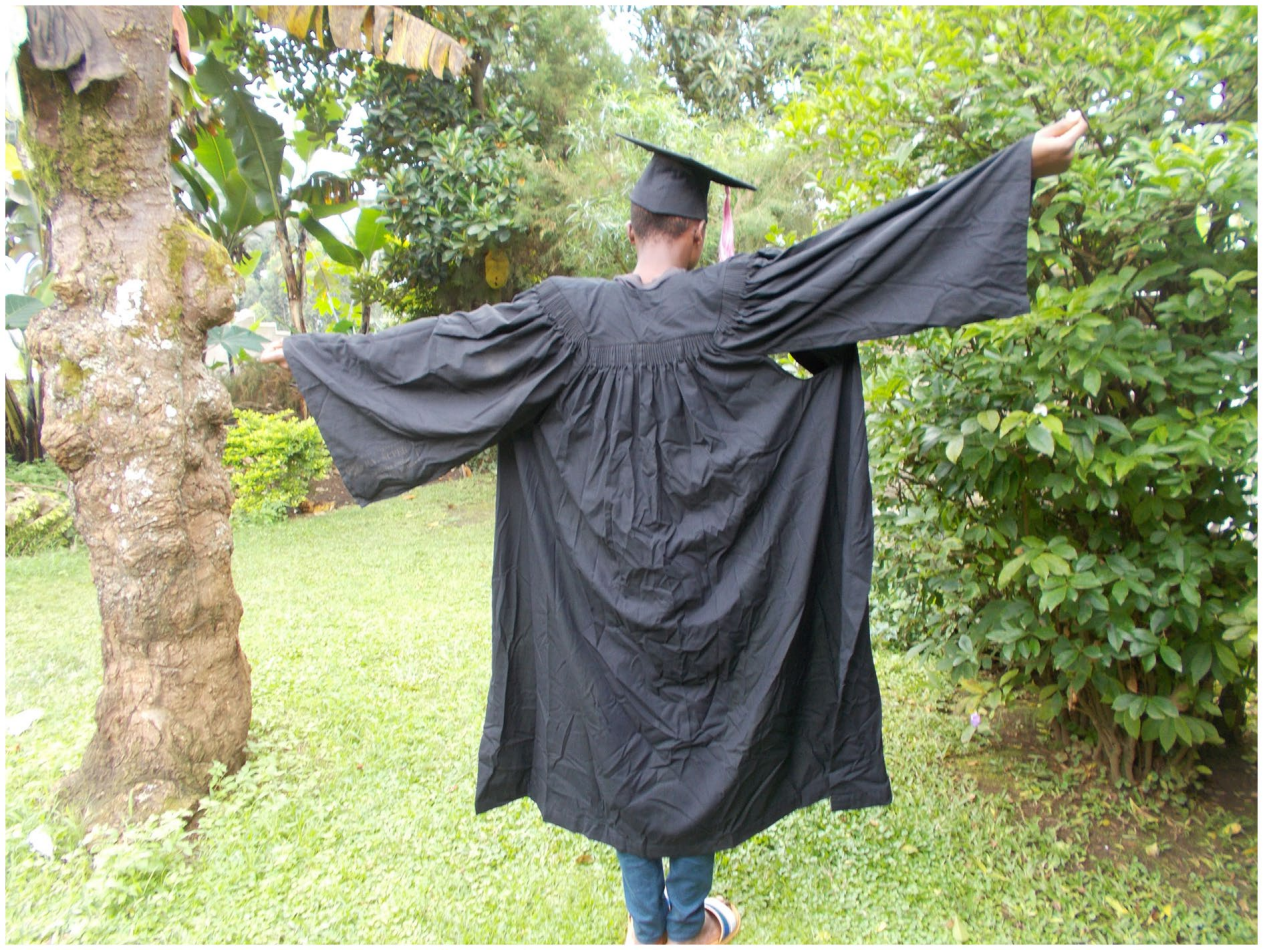
“Being HIV positive cannot stop me from achieving the things I have always wanted in my life.” Josh, 20-year-old boy.

The interconnectedness between resources like education and tensions in power and control also featured prominently in Josh’s photos and narrative, as he spoke at length about the importance of “looking beyond your situation”. The following quote and photo (Figure 3) are illustrative:

"Being HIV positive cannot stop me from achieving the things I have always wanted in my life. I always wanted to study and become a doctor and I know that this dream is about to come true because I am now studying at (name of institution) to get my diploma in clinical medicine. My studies have made me understand HIV and how to live a healthy life. My life has changed so much that those who saw me a few years ago might not recognize me. I also want to use this knowledge to help other young people with HIV.” – Josh, 20-year-old boy

### 3.4. Cultural adherence

Participants emphasized the importance of “trying to live a normal life” and being able to “achieve what society does not expect”, which they collectively identified as important themes. These themes relate to tensions in cultural adherence, which is about “adherence or opposition to one’s local and/or global cultural practices, values and beliefs” (Ungar et al., 2007, p. 299).

Cultural values and beliefs play a vital role in shaping the norms and expectations that exist within a community. According to the participants, young people in their communities are generally expected to be busy, productive and contributing to their family, community and society. YLWH are mostly seen as unable to achieve this, because of their serostatus. They are thought of as weak, suffering and vulnerable, which risks relegating them to the margins of society.

While participants experienced that it may be more difficult for them to act, interact and fulfil the roles set out by cultural practices, values and beliefs, they also spoke at length about how strengthened they felt when they were able to live up to the prevailing norms and expectations, and feel that they could live a “normal life”. This is illustrated in Figure 5 and the following quotes:

**Figure 4:**
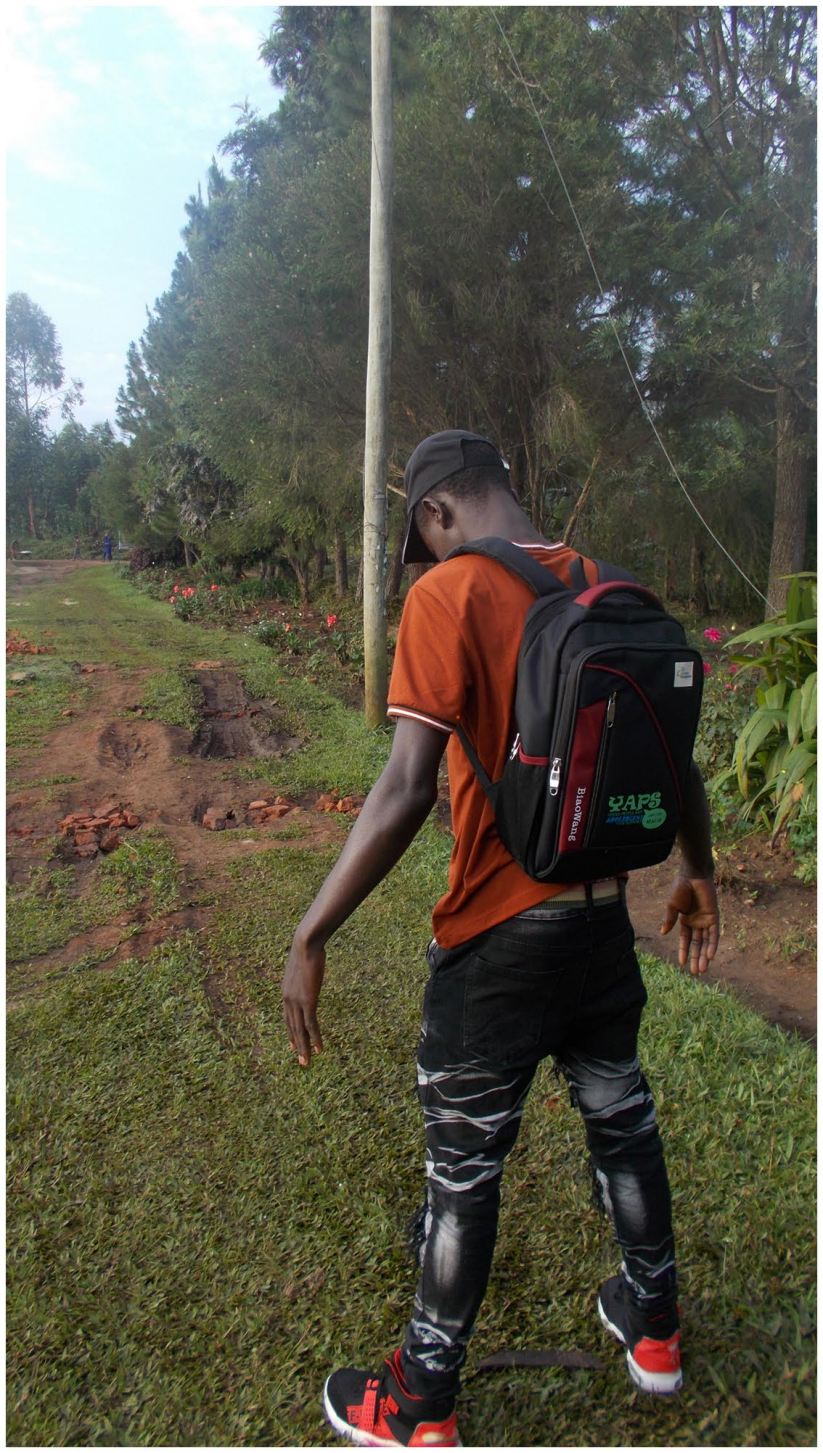
“I try to live my life like other youth even if I have HIV” King, 17-year-old boy.

“I try to live my life like other youth even if I have HIV. I am always smart, I buy for myself nice clothes and I am always happy. I realized that I can choose to enjoy life or to live a miserable life. I thank people like doctors who have counselled me and advised me to work hard and not to wait for other people to give me help.” – King, 17-year-old boy

“I took a photo of my school. At school, I am always busy concentrating on my studies because I know I will have a good future if I study. I don’t care what is in my body or what other people say about me because if I concentrate on that, it will not change anything. My mother taught me to ignore people and focus on what is good for me.” – Junior, 14-year-old boy

Rather than adhering to the prevailing expectations about YLWHA and accepting the disqualification, disempowerment and even dehumanization that this entails, YLWH in this study plainly opposed to this and aimed to exemplify alternative representations that can change these cultural practices, values and beliefs around HIV and the young people living with it.

Resources related to resolving the tension of cultural adherence is what the youths thematized as “being busy”, “having future plans”, and “focusing on set goals”. Resources were seen as meaningful if they strengthened them to pursue meaningful roles and occupations in relation to socio-cultural views and ideals. Opportunities for them to keep busy and experience progress towards set goals or aspirations, e.g., through (informal) education or (voluntary) work, were valued because they allowed them to conform to practices, values and beliefs generally held for youths and not to feel different. For them, this meant working hard to achieve something, and even feeling the urge to work harder than others in order to surpass prevailing academic, economic, and cultural expectations and be someone in their society.

### 3.5. Relationships

The photos and narratives further showed appreciation of supportive relationships, often forged amidst myriad disempowering encounters. Relationships in and of themselves turn out to be resourceful in dealing with challenging situations, but are also instrumental in obtaining access to other resources (Ungar et al., 2007). The youths identified the following related themes: “peer support”, “peer role models”, “family support”, and “support from healthcare workers”, hence referring to both informal and formal supports.

**Figure 5:**
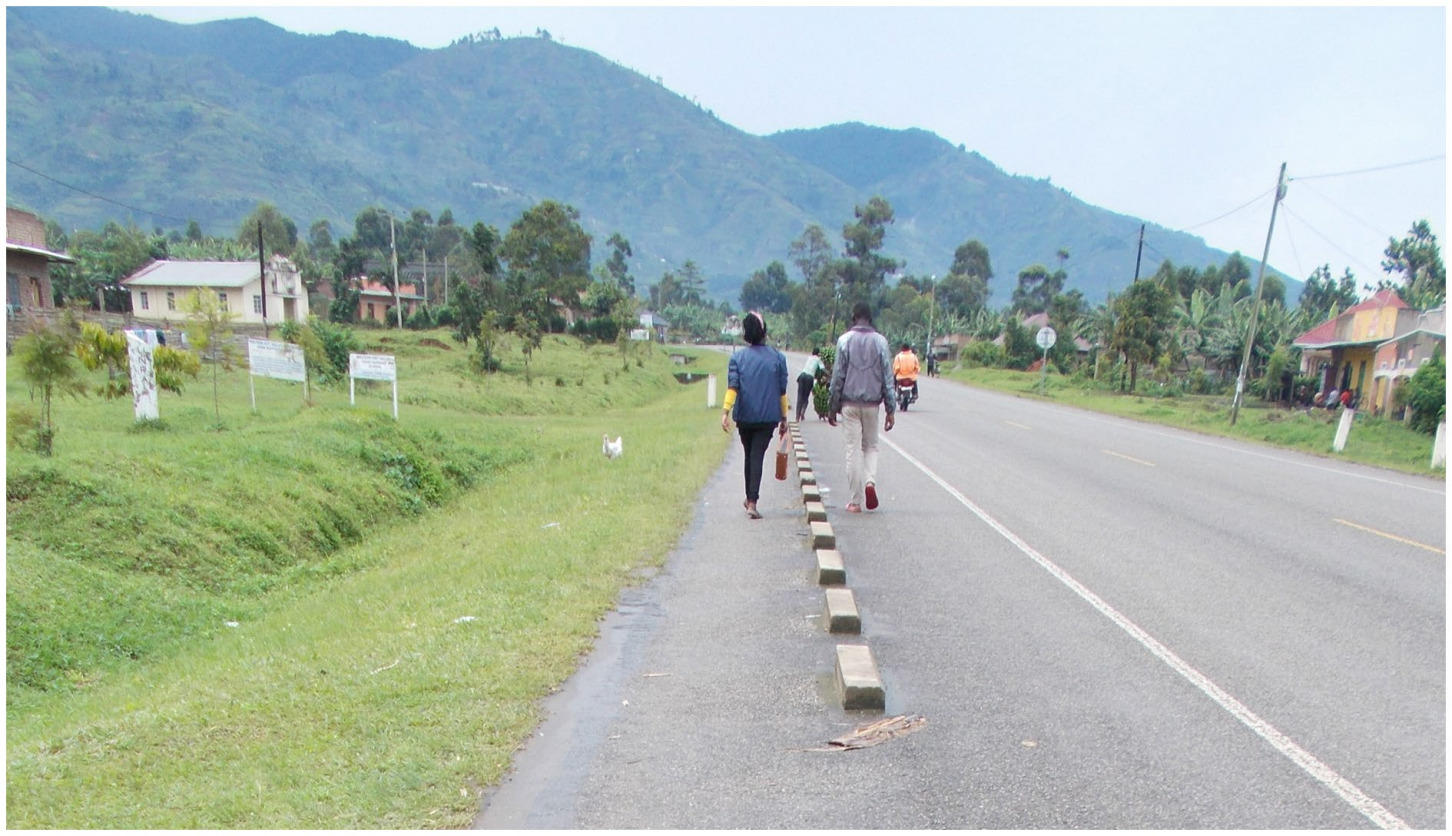
“I thank them very much because I know that I’m alive because of them” Josh, 18-year-old-boy.

Role models empowered them to develop a positive outlook on the future and reinforced their own efforts and capacities to experience feelings of wellbeing. Family members, neighbors and friends were also able to foster resilience in the face of HIV-related challenges, as evident in the following quotes and Figure 5:

“I took this photo to show my mother’s love. From the moment my father died in 2015, our mother has been there for me and my siblings. She cares for all of us by feeding us and protecting us equally, just like this hen takes care of her chicks (on the photo). It has not been easy but at least I know there is someone who cares for me.” – Brenda, 15-year-old girl

“This is a photo (Figure 5) of me walking with a friend. I found out that on this earth you need someone with whom you can take a journey of life. That person should also be positive and should be able to hold your hand and support you through some difficult times. When I lost my mother, the only parent I had, I felt very bad and even stopped taking my ARVs (antiretroviral medicine). If my close HIV-positive friends had not comforted me, I would also be dead now. I thank them very much because I know that I’m alive because of them.” – Josh, 18-year-old boy

The participants emphasized the importance of reciprocity in relationships and thematized “being of value to others” and “getting recognition”: being able to participate and contribute, feeling needed and appreciated, and experiencing belonging. Such relationships were seen as resourceful in nurturing and maintaining a positive, coherent sense of self and thus intersect with identity tensions.

### 3.6. Cohesion

Participants’ photos also spoke to managing tensions in cohesion or “the convergence of one’s sense of responsibility to self and a philosophy of duty to one’s community’s greater good” (Ungar et al., 2007, p. 298). Cohesion is about “feeling of being a part of something larger than one’s self socially and spiritually” (Ungar and Liebenberg, 2011, p. 136). The following quotes are illustrative:

“I enjoy playing football with my friends at school and in the village. Whenever I am playing football, I enjoy and forget about all the worries I may have. I am also a very good striker and my team cannot win without me. When they are going to play a match or for training, they have to call me.” – Josh, 18-year-old boy

**Figure 6:**
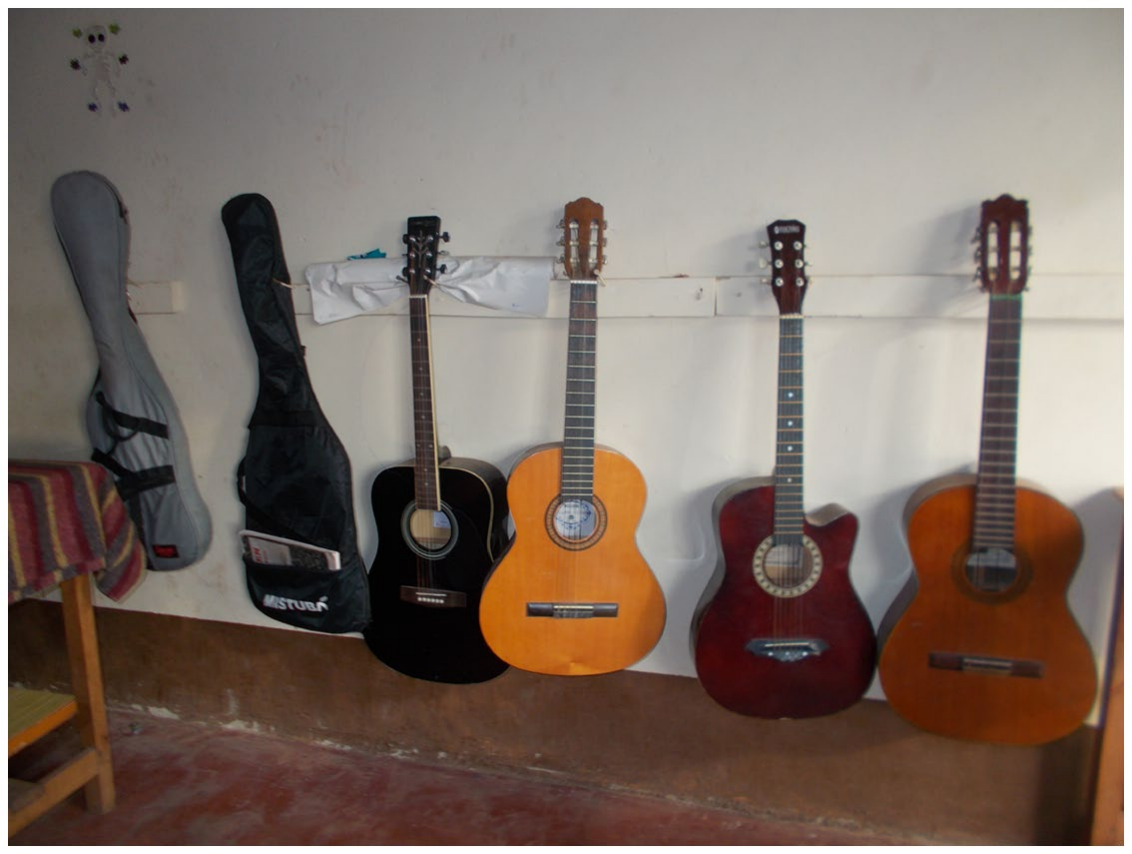
“I got many friends in the community who wanted me to teach them how to play band instruments. Playing in the band took away all the fears I had.” Inbox, 19-year-old boy.

“These (Figure 6) are guitars in our music room. When I joined the band, I learnt to play many instruments, and young people and adults in the community started to admire me. I felt very good and important because they would even shout my name during performances. I got many friends in the community who wanted me to teach them how to play band instruments. Playing in the band took away all the fears I had.” – Inbox, 19-year-old boy

Participants also valued supportive and safe spaces in the community and in youth services that make them feel welcome, accepted and valued, regardless of their serostatus. Such spaces are seen as important in managing tensions in cohesion and also in identity. In her photos and narrative, Maggie referred to the “youth-friendly health centre” as such a safe and supportive space for her and other YLWH:

“I took a photo of part of the poster for our clinic. In my language (rutooro), ‘*irwarro*’ means clinic. This clinic is like home. The nurses are nice to me and other youths who come here. Whenever I come to the clinic, I find other youths and we play different games together. I do not want to miss days of coming to the clinic. Because we have different games here, other children in the community come here to play with us and they have become our friends. They do not discriminate against us even if they know that we have HIV.” – Maggie, 19-year-old girl

### 3.7. Social justice

Participants’ accounts of how they try to manage the tensions mentioned above appear to be strongly intertwined with individually and collectively experienced injustices. Such injustices affect, among other things, their sense of identity, cohesion, power and control, and deeply permeate their daily lives. These young people are clearly trying to forge a position in which they can experience social justice.

According to Ungar et al. (2007, p. 300), “social justice is a theme that captures experiences of prejudice and dynamics of sociopolitical context encountered individually, within one’s family, in one’s community and culture, as well as experiences of resistance, solidarity, belief in a spiritual power, and standing up to oppression.”

Spirituality, for instance, figured in participants’ photos and narratives as a resource in situations of social injustice that create tensions in identity and cohesion. Maggie’s quote is illustrative:

“This is a church close to our home. Whenever I feel sad or when someone has said bad things to me, I go and sit in this church and pray. It gives me hope that I have a father in heaven even if my father on earth died. I always feel good when I go to church and I pray. The Bible says: ‘Cast your burdens to Jesus for he cares for you.’” – Maggie, 19-year-old girl

The theme of social justice is further about finding a meaningful role in the community and about social equality (Ungar and Liebenberg, 2011). The themes identified by the participants as “constantly seeking better life” and “persevering” against all odds strongly attest to their perpetual quest for justice and for experiencing well-being and inclusion amidst the disadvantaging and disempowering forces in their social ecologies. These themes also reverberate the conceptualization of resilience as “a condition of becoming better” (Ungar et al., 2007, p. 301).

**Figure 7:**
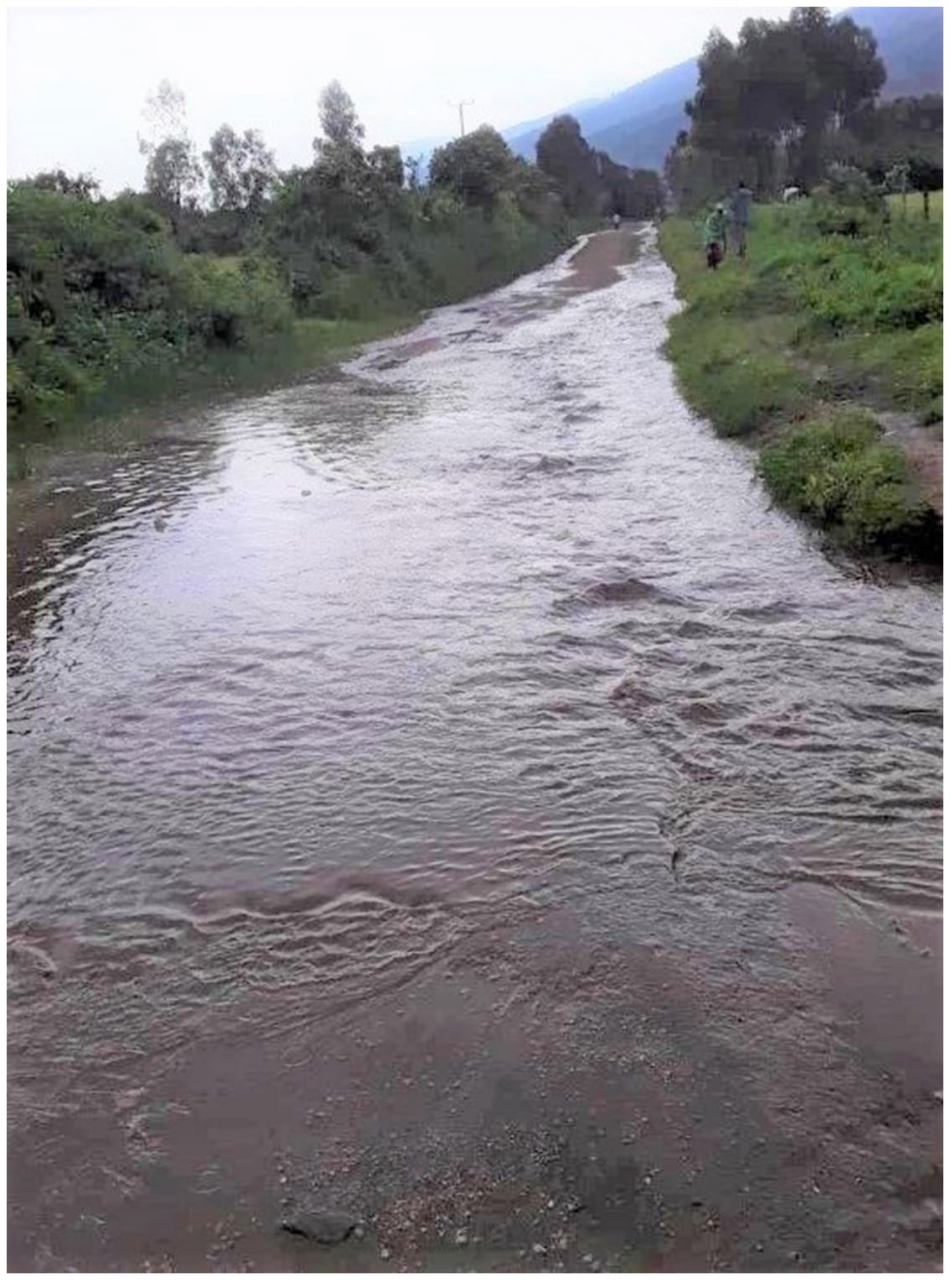
“I do not know what lies before me but I now it will be better.” Abooki, 19-year-old boy.

This directs our gaze towards the small but significant daily acts and activities undertaken by the participants, their significant others and youth services to respond to, resist, refute and contradict contextually enshrined expectations, representations and approaches to YLWH. This is illustrated by the quote of Abooki, who stated:

“I have allowed myself to be on the move like this water in the stream (Figure 7). I do not stay in one position because I have to continuously look for people and things that can help me in my situation. I have to look for information, I have to look for money, and I have to look for caring people. I also have to move away from bad situations, from people who abuse me, from things that remind me of the past and from those who discriminate me. I do not know what lies before me but I know it will be better.” – Abooki, 19-year-old boy

Throughout the study it was recognised that these young people had become used to perceiving themselves in a disempowering way and it was initially difficult for them to appreciate their own capacities and social ecological resources. Being guided to do this was reported to be very empowering, both individually and as a group, as the process and advanced insights seemed to be an antidote to the structural disempowerment they had bought into. The following quotes illustrate the importance of a social justice approach to resilience research:

"When people come to talk to us, we people with HIV, they usually ask us what problems we have. Many young people with HIV are used to only seeing problems. Sometimes we think that if we tell people our problems, they will help us or give us something. But with what we have talked about now, I see that we have the ability to live like others, using our own energy, using the things and people around us, and not just to make others feel sorry for us, that 'oh sorry this one has HIV and he is going to die'. Because we are not going to die any time soon, we are going to die like all other people even those without HIV. We, young people with HIV, have to change our thinking and then other people will change the way they look at us”. – Inbox, 19-year-old boy

"I have never had a discussion like this, even in our meetings and conferences of the peer support group. We always talk about things that affect us, how to comply with our treatment and how to avoid stigmatisation. I think it is important to start being happy about ourselves, and realize that we can live like other people, and even better. Someone can even ask you 'what do you like about yourself?' and you say ‘nothing’. But there are many things we can be happy about. I now have a child, but many women are infertile, even those without HIV. We have heard what the young people here can do that those without HIV cannot do. We must thank God for these things and stop thinking that we have no use in this world.” – Passion, 17-year-old girl

## 4. Discussion

This study sought to explore YLWH’s perceptions and representations of social ecological resources that enable them to experience feelings of well-being and contribute to resilience in the context of HIV-related challenges. Photovoice methodology was used to gain an insider’s perspective on participants’ everyday realities and lived experiences, and to provide them with a platform for self-representation. Drawing on their expertise-by-experience, YLWH scrutinized their daily lives in search of meaningful events and experiences that they associate with resilience.

These everyday experiences – while seemingly trivial or mundane – are crucial to deconstructing and understanding how HIV infiltrates all spheres of life, how YLWH and their contexts deal with this and how this affects YLWH’s sense of wellbeing. In addition to demonstrating that HIV generates challenges and evokes social injustice in many subtle and not-so-subtle ways, this research adds to the body of knowledge on youth resilience in Sub-Saharan Africa by documenting multisystemic resilience resources for young people living with HIV in western Ugandan communities.

Our findings resonate with Ungar (2008)’s cross-cultural insights into the tensions that young people must navigate to experience resilience. Their daily efforts to respond to, resist, refute and contradict harmful direct and indirect effects of HIV on material resources, identity, power and control, cultural adherence, relationships, cohesion, and social justice are richly documented in their photographs and narratives.

Notwithstanding these global characteristics of resilience, the study’s findings also illustrate the contextual entanglement of challenges and resources, as well as what are considered positive outcomes for these youths. The emic perspectives on resilience and the constitutive everyday supports in the face of HIV affirm the need for a contextually affiliated approach, both for how resilience is understood and conceptualized (Vindevogel et al., 2015) and for identifying the constellation of multisystem resources that matter (Theron and Van Breda, 2021a).

The study shows that how young people experience and navigate tensions in resilience reflects how HIV has come to be understood and addressed in their context. This relates to local philosophies, based on the ontological and normative systems in place (Flint, 2018), and intersects with the social, cultural, political, religious and historical dimensions of that locality. In Sub-Saharan African countries like Uganda, strong cultural and religious systems influence social constructions of YLWH (Odokonyero et al., 2022) and, in turn, the support available to them. As such, contextual factors and dynamics affect resilience processes and outcomes (Crowley et al., 2021). Many of the resources identified by the youths in this study appear to support their struggle to show humanness, relate communally and restore or gain social harmony, which is the highest good from an Afro-communitarian point of view (Flint, 2018).

YLWH are bound to experience multiple forms of social injustice that undermine their ability to live up to these values and norms. Such injustices arise in interaction with both informal and institutional layers of the social ecology and lead to dehumanization, seclusion and discord among other consequences (Kimera et al., 2020b). The resources documented in this study can be understood as significant in countering individually and collectively experienced injustices such as the social devaluation, discrimination and exclusion of YLWH in families, communities, schools and workplaces, among others.

The resources that support youths in navigating and resolving such HIV-related challenges and tensions in resilience equally stem from their social and institutional contexts. These resources are mainly located in families, peer groups, schools, health centers and communities. Thus, this study confirms the conceptualization of resilience as the collective capacity of the individual and the social ecology (Ungar, 2008; Theron and Van Breda, 2021a). Furthermore, this study shows that youth services, HIV clinics/health centers and schools, among other institutionalized supports, have the potential to be supportive spaces that empower YLWH and mobilize resources in the wider community for this purpose, if they integrate resilience perspectives in their daily operations and intervention programming. This confirms findings from earlier studies on YLWHA in Uganda (Nabunya et al., 2020).

Macrolevel systems, such as local and national government or religious institutions, were not explicitly highlighted by participants, even though the body of literature on youth resilience has convincingly shown that such macrosystems can play an important role in co-constructing resilience pathways and strengthening supports within and across other systems (Shevell and Denov, 2021). There may have been photographs that participants could not take or stories that they could not tell, simply because of a lack of resources such as an effective anti-stigma program or legal system to tackle discrimination.

In addition, the multisystemic supports highlighted by YLWH in this study appear to be highly incidental and situational, not widely available or structurally embedded. Statements such as “I live because of them” and “at least I know there is someone who cares for me” speak to the overly challenging and structurally disadvantaged context in which YLWH find themselves. While such incidental experiences of resilience can be very powerful and meaningful for young people, as this study shows, they risk being hampered by the unavailability of structural resources and even existing structural barriers to resilience in society (Wessells, 2018). This finding confirms the earlier identification of policy and programmatic gaps in the HIV care continuum in Uganda (Obare et al., 2011).

This study therefore endorses the call to strengthen a nested system of social-ecological resources and resilience processes around youth, but also to address the injustices that arise from these very social ecologies and continue to jeopardize the wellbeing of many young people in Uganda and other settings (Hart et al., 2016; Crowley et al., 2021; Wessells, 2021). Policy makers and practitioners working to support YLWH should take heed of the multisystemic sources of resilience as well as the current lack of structural support/existing structural barriers to it. Such macrolevel actors are in a position to address systemic and pervasive injustices and to minimize the ensuing risks and challenges for YLWH, alongside structurally embedding resources that enable them to overcome these and even thrive while living with HIV.

Building strong ecologies of resilience should also be at the forefront of the global HIV/AIDS-agenda if we are to achieve the Sustainable Development Goal of ensuring healthy lives and promoting well-being for all at all ages. This requires moving beyond the dominant bio-medical approach to a holistic approach that considers the full scope of biomedical, psychological, social, and societal challenges (Goodenow and Gaist, 2019), grafted upon the understanding of the complex dynamics between health and society. Recent studies suggest that approaches from the social sciences are essential to understand and address the individual, interpersonal, community, societal, and structural factors influencing HIV/AIDS-related disparities worldwide (Goodenow and Gaist, 2019; Odokonyero et al., 2022).

In addition, our study shows that emic perspectives, and in particular expertise-by-experience, should inform this approach in order to be in sync with the often complicated life situations in which young people try to manage their health and experience wellbeing. This requires acknowledging YLWH as meaning makers, knowledge creators and change agents, and leveraging their potential to advance evidence, interventions and policy development (Suffla et al., 2012; Fournier et al., 2014). Participatory methods such as photovoice have been underutilized in the HIV field, but are proving to provide powerful insights that can catalyze social action and change (Wang and Burris, 1997). This study’s findings should be interpreted against the backdrop of some limitations. Recruitment of participants from an existing peer support group may have biased the results, as these young people already have experience of health care and peer support and may therefore have access to more resources or have developed a more positive view of social-ecological support. The study did not analyze the positionality of the participants or the characteristics of their social ecologies. It is possible that social ecologies respond differently or mobilize more/different resources depending on these factors. Furthermore, experiences of resilience are strongly influenced by spatial and temporal dynamics (Ungar et al., 2007). Future research can address how experiences of resilience evolve as context changes over time. In addition, a thorough gender analysis could reveal differences between boys and girls.

Only when the intersecting sources and barriers to resilience within and across all social-ecological levels are made visible, through this and future research, can they be addressed and can pathways be illuminated for research, policy and youth service programming to support resilience of young people living with HIV in western Ugandan communities.

## Data availability statement

The raw data supporting the conclusions of this article will be made available by the authors, without undue reservation.

## Ethics statement

The studies involving human participants were reviewed and approved by Uganda National Council of Science and Technology (UNCST); Institutional Review Board of The AIDS Support Organization (TASO) in Uganda; Commissie Medische Ethiek Vrije Universiteit Brussel; Written informed consent to participate in this study was provided by the participants' legal guardian/next of kin. Written informed consent was obtained from the individual(s), and minor(s)’ legal guardian/next of kin, for the publication of any potentially identifiable images or data included in this article.

## Author contributions

SV contributed to the conception and design of the study, the analysis and interpretation of data, and the reporting and publishing of the findings. EK contributed to the design of the study, the acquisition, analysis, and interpretation of data, and the reporting and publishing of the findings. All authors contributed to the article and approved the published version.

## Funding

This study was funded by the Fund for Applied Scientific Research of HOGENT University of Applied Sciences and Arts Ghent.

## Conflict of interest

The authors declare that the research was conducted in the absence of any commercial or financial relationships that could be construed as a potential conflict of interest.

## Publisher’s note

All claims expressed in this article are solely those of the authors and do not necessarily represent those of their affiliated organizations, or those of the publisher, the editors and the reviewers. Any product that may be evaluated in this article, or claim that may be made by its manufacturer, is not guaranteed or endorsed by the publisher.
